# Deliberate practice: The cornerstone of microsurgical therapy

**DOI:** 10.1002/cap.70055

**Published:** 2026-04-15

**Authors:** Diego Velásquez‐Plata, Rino Burkhardt

**Affiliations:** ^1^ Private Practice Fenton Michigan USA; ^2^ University of Michigan School of Dentistry Ann Arbor Michigan USA; ^3^ Private Practice Zurich Switzerland; ^4^ Center of Dental Medicine University of Zurich Zurich Switzerland; ^5^ Prince Philip Dental Hospital, The University of Hong Kong Hong Kong China

**Keywords:** clinical competence, learning curve, microsurgery, simulation training

## Abstract

**Background:**

Effective use of the operating microscope in periodontal and implant procedures demands the acquisition of specialized skills. Clinicians must adapt to high‐magnification workflows, requiring exceptional precision through bimanual instrumentation. This perspective article aims to explore the principles of deliberate practice by outlining its defining components and core characteristics and providing actionable guidance for the implementation of this approach.

**Methods:**

A conceptual analysis was conducted to evaluate the role of incorporating core principles of deliberate practice as a mechanism for developing and enhancing skills within microsurgical training workflows.

**Results:**

Adopting deliberate practice can significantly enhance skill development and accelerate the proficiency timeline in advanced periodontal and implant‐related clinical protocols.

**Conclusions:**

The essential components of deliberate practice are identified and defined, and practicable frameworks for applying this approach are illustrated. Its benefits and limitations are also discussed.

**Key Points:**

- Deliberate practice is a structured‐goal oriented approach designed to maximize skill development.
- Continuous feedback serves to refine methodologies, prevent stagnation, and ensure execution aligns with expert benchmarks of excellence.
- Sustained effort and mental engagement are the outcomes of consistency, dedication, and perseverance through periods of intense focus.

**Plain Language Summary:**

An operating microscope is used in periodontal and dental implant related procedures mainly because it allows dentists to see small details with great accuracy. Working under high magnification demands special training since small hand movements must be extremely precise. In order to overcome these challenges, there is a training approach called deliberate practice that can make the process more effective. This article explains how this method helps dentists improve their microsurgical skills, ultimately leading to better care and outcomes for patients.

## INTRODUCTION

In the Victorian era, exceptional skills were largely viewed as innate manifestations of mental prowess, predominantly determined by heredity. The role of environmental factors in developing abilities was considered minimal, with “nature” firmly trumping “nurture”.^1^ Since then, the science of expertise has undergone a significant evolution in its understanding of high performance across diverse domains. This transformation encompasses fields such as sports, music, games, medicine, and education, as well as specialized areas like typing, programming, and trading. Modern research now recognizes a more nuanced interplay between genetic predisposition and environmental influences in shaping extraordinary abilities.^2^, ^3^, ^4^, ^5^, ^6^

The late Anders Ericsson and his team of researchers dedicated themselves to unraveling the mystery of expert‐level performance.^7^, ^8^, ^9^ Their focus shifted from innate talent to the crucial role of specific training methods in cultivating expertise. Through decades of observational and interventional studies, Ericsson's group illuminated the conditions and environments that foster domain‐specific mastery. Their groundbreaking work unveiled the mechanisms underlying the development of exceptional skills, challenging long‐held beliefs about the primacy of natural ability. In the early 1980s, Ericsson and Chase conducted a groundbreaking study on memory improvement that revolutionized our understanding of cognitive enhancement.^10^ Their research, which focused on a single participant's ability to recall random digits, identified several key factors crucial to expanding memory capacity. Chief among these were structured practice techniques coupled with immediate feedback, implemented over sustained periods of intense focus. This methodical approach led to remarkable improvements in cognitive abilities previously thought to be fixed. The study's impressive results not only challenged long‐held beliefs about the limits of human memory but also laid the groundwork for further investigations into cognitive enhancement across various domains.

In 1993, Ericsson et al. introduced the concept of deliberate practice in a paper titled “The role of deliberate practice in the acquisition of expert performance,” defining it as a series of activities structured to improve the levels of current performance.^11^ These activities target identified weaknesses, and performance outcomes are closely monitored to provide feedback geared towards achieving further improvements. Deliberate practice can only be sustained for a limited time since it requires extraordinary effort, and it is neither easy nor enjoyable by nature.

In the realm of microsurgery, the development and maintenance of skills are paramount to ensuring optimal performance during patient care. Deliberate practice, with its emphasis on well‐defined and specific goals, serves as a cornerstone for beginners, intermediate practitioners, and experts in honing their surgical skills. By designing targeted activities that address perceived weaknesses, instructors can maximize the efficiency of time and effort invested during practice sessions, effectively overcoming these limitations.

**FIGURE 1:**
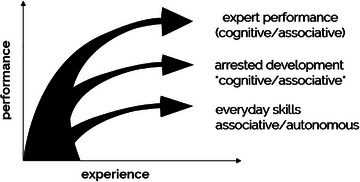
Deliberate practice in the acquisition of expert performance. The entry level of performance is to create an autonomous stage of execution when dealing with everyday tasks that demand basic skills. Automaticity (arrested development) emerges through the cognitive and associative phases of skill acquisition, ultimately enabling task performance with minimal effort. Expert performers will break free from automaticity by seeking higher levels of control of their performance, staying in the cognitive and associative phases. Adapted from Ericsson (2018).^12^

Pushing practitioners beyond their comfort zones creates invaluable opportunities for growth, challenging them to execute tasks that stretch the boundaries of their current abilities. These intensive training regimens demand unwavering focus and concentration, characterized by their high intensity and the need for constant, refined repetitions. Through this rigorous approach, microsurgeons can continually elevate their skills, ultimately translating to superior patient outcomes (Figure 1).

The purpose of this perspective article is to expand the concept of deliberate practice as it applies to microsurgical applications in periodontics and implant dentistry.

## COMPONENTS OF DELIBERATE PRACTICE

### Structured approach

Deliberate practice in microsurgery adheres closely to a well‐defined script that has been curated by a microsurgical instructor with clear goals per practice session. Each training period focuses on a specific set of skills to be developed, managing a unique weakness that is hindering advancement, or the refinement of already established competencies.

For example, Video 1 demonstrates an exercise focused on optimizing the fluidity of surgical needle transfer using coordinated bimanual instrumentation—a fundamental movement pattern critical to real‐world surgical applications. The setup features closely spaced plastic hoops, requiring trainees to alternate between needle holders and tissue holders to pass needles through the hoops' openings. This forces dynamic adjustments in instrument angulation and grip positioning, with timed trials providing quantitative metrics for performance evaluation (Video 1).

**VIDEO 1:** Needle transfer exercise. Hand‐eye coordination during bimanual instrumentation is a technical surgical skill that must be learned, developed, and enhanced with deliberate training. This exercise focuses on holding and releasing a needle accurately and in a timely manner while thrusting it through the opening of small beads that closely match the needle diameter. The goal is to minimize unnecessary needle manipulation by anticipating the needle orientation needed to go through each bead. See accompanying video file.

### Effortful and challenging

Not all practice engagements are created equal (Table 1). When immersed in a deliberate practice session, the trainee is being pushed beyond the comfort zone. This is one of the key differentiating factors when compared to a practice strategy that involves repetition without a clear goal or feedback in response to demands generated by external factors, also known as naïve practice.^13^ Contrary to a state of “flow” in which a trainee is joyfully immersed in an activity with low attention demands,^14^ deliberate practice is mentally strenuous in nature, requesting full focus and concentration on the task at hand. By identifying challenges and laying out strategies to learn new skills and face difficult steps related to a particular technical clinical application, personal growth, increased confidence, strengthened resiliency, and enhanced creativity are forged. Mastering needle handling in microsurgery presents unique challenges, particularly when it comes to precisely inserting and advancing microsurgical needles within delicate structures and confined spaces. To refine these skills, one effective and structured practice exercise involves fingertip‐driven needle rotation. This technique uses small plastic jewelry beads placed on the individual drupelets of aggregate fruits, such as blackberries or raspberries, as a training medium. The exercise is both effortful and challenging, simulating the precision required in microsurgical procedures while fostering dexterity and control (Video 2).

**TABLE 1:** Comparison of key features of practice types. Adapted from Ericsson (2016 and 2019).^13^, ^15^

			Feedback		
Practice type activities	Expert guidance	Defined goals	Delivery	Specificity	Effectiveness
Deliberate	Yes‐ individual	Customized	Immediate‐Expert‐led	Highly detailed and task‐specific	High
Purposeful	Yes‐ sporadic	Specific	Delayed‐Self/peer	Generalized	Moderate
Structured	Yes‐group	Generic	Delayed‐Self/peer	Generalized	Moderate
Naive	No	None	None	None	Low

**VIDEO 2:** This exercise's goal is to practice finger‐tip‐driven needle rotation in confined spaces and working with delicate structures. A micro jewelry/craft bead is placed on top of one of the drupelets (drupelets are individual bumps of aggregate fruits such as blackberries and raspberries), and it is secured with four individual sutures utilizing a small needle (6 mm) with a 9‐0 or 10‐0 suture. See accompanying video file.

### Continuous feedback

Receiving continuous feedback is a powerful tool for improving performance, as it enables trainees to recognize their strengths and gain a deeper understanding of the areas where they can improve. This heightened self‐awareness serves as the foundation for refining their approach, ensuring adjustments are purposeful and effective, ultimately driving sustained growth over time.

Moreover, learning is significantly enhanced when feedback delivers tailored insights that address specific needs. Sharing both performance knowledge (process) and results knowledge (outcomes) delivers valuable feedback for skill development and refinement.^16^, ^17^ It fosters open and constructive communication between instructors and trainees, creating a collaborative environment that encourages growth. Continuous feedback also facilitates regular evaluation and alignment of goals, ensuring efforts remain focused and meaningful. Additionally, it reinforces accountability by consistently tracking progress, motivating trainees to stay committed to their development journey.

Setting measurable goals offers clarity and focus by providing quantifiable objectives, making it easier to determine when those goals have been successfully achieved. When trainees reach their targets, they experience a sense of accomplishment that fuels motivation to tackle future objectives, creating a positive cycle of achievement and growth. Clearly defined metrics and milestones enable progress tracking and foster accountability, ensuring that any unmet goals can be addressed proactively.^18^ In such cases, tailored strategies can be developed to overcome obstacles and help the trainee realign with their objectives. Additionally, identifying what strategies are effective and what approaches fall short serves as an invaluable tool in driving continuous improvement and sustained progress.

**FIGURE 2:**
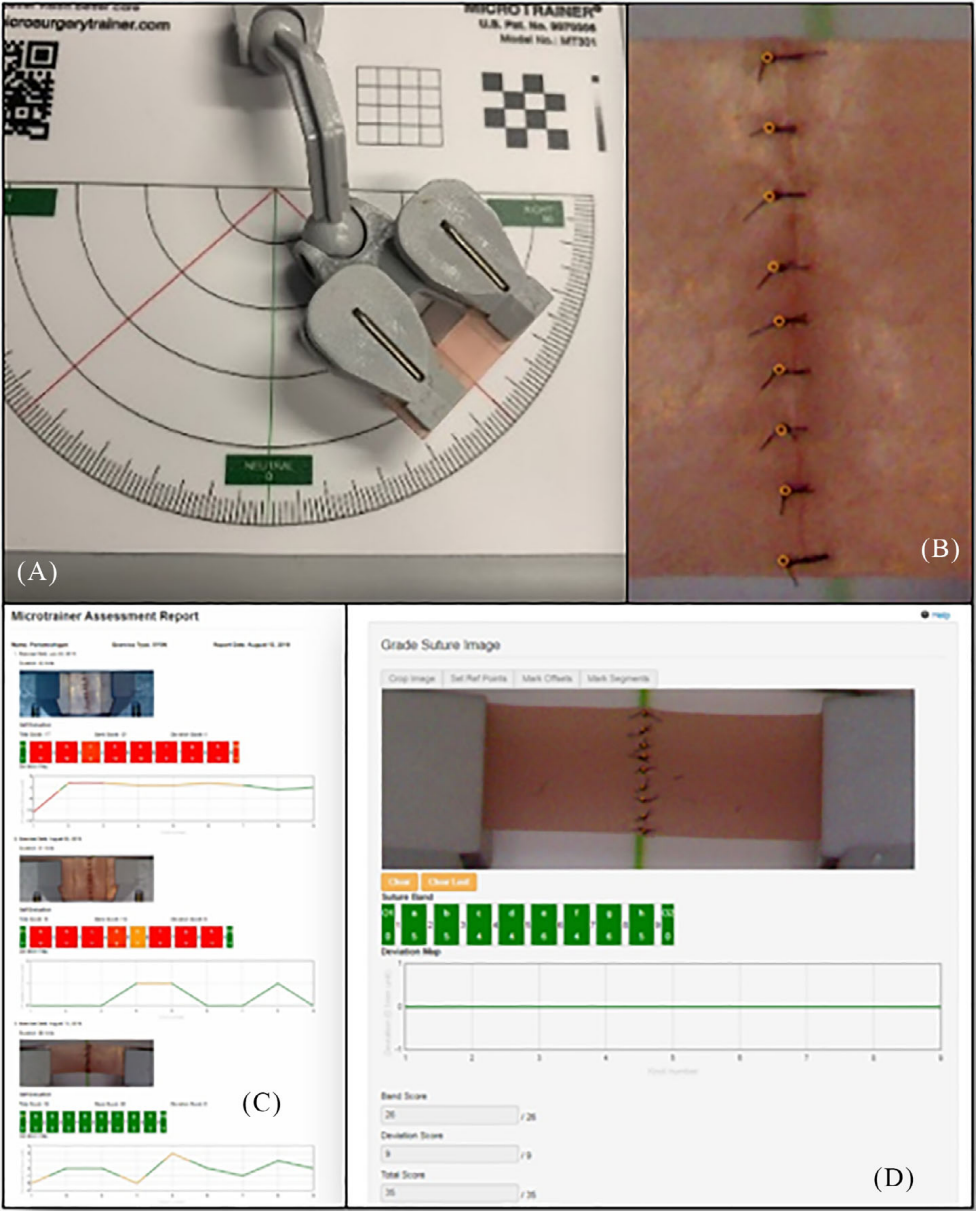
Suture simulator platform. (A) Training platform with a movable arm to facilitate practice in different horizontal and vertical positions. (B) Suturing exercise with captured data to assess symmetry. (C and D) Cloud‐based application that provides immediate performance feedback and tracks progress over time.

Constant evaluation of progress is another cornerstone of deliberate practice since it allows identification of areas of weakness, tracking progress over time, and ensuring that practice remains focused and efficient. Without constant evaluation, falling into a comfort zone of stagnation is a risk that will inevitably lead to reaching a plateau and inhibit further progress in learning and skill acquisition. A meditech startup has created a simulator platform^1^ that allows the trainees to develop microsurgical suturing skills, accompanied by a cloud‐based application that provides immediate performance feedback and tracks progress over time. This self‐guided approach to training is useful once foundational skills are in place and direct, in‐person instruction is not readily available (Figure 2 ).

**FIGURE 3:**
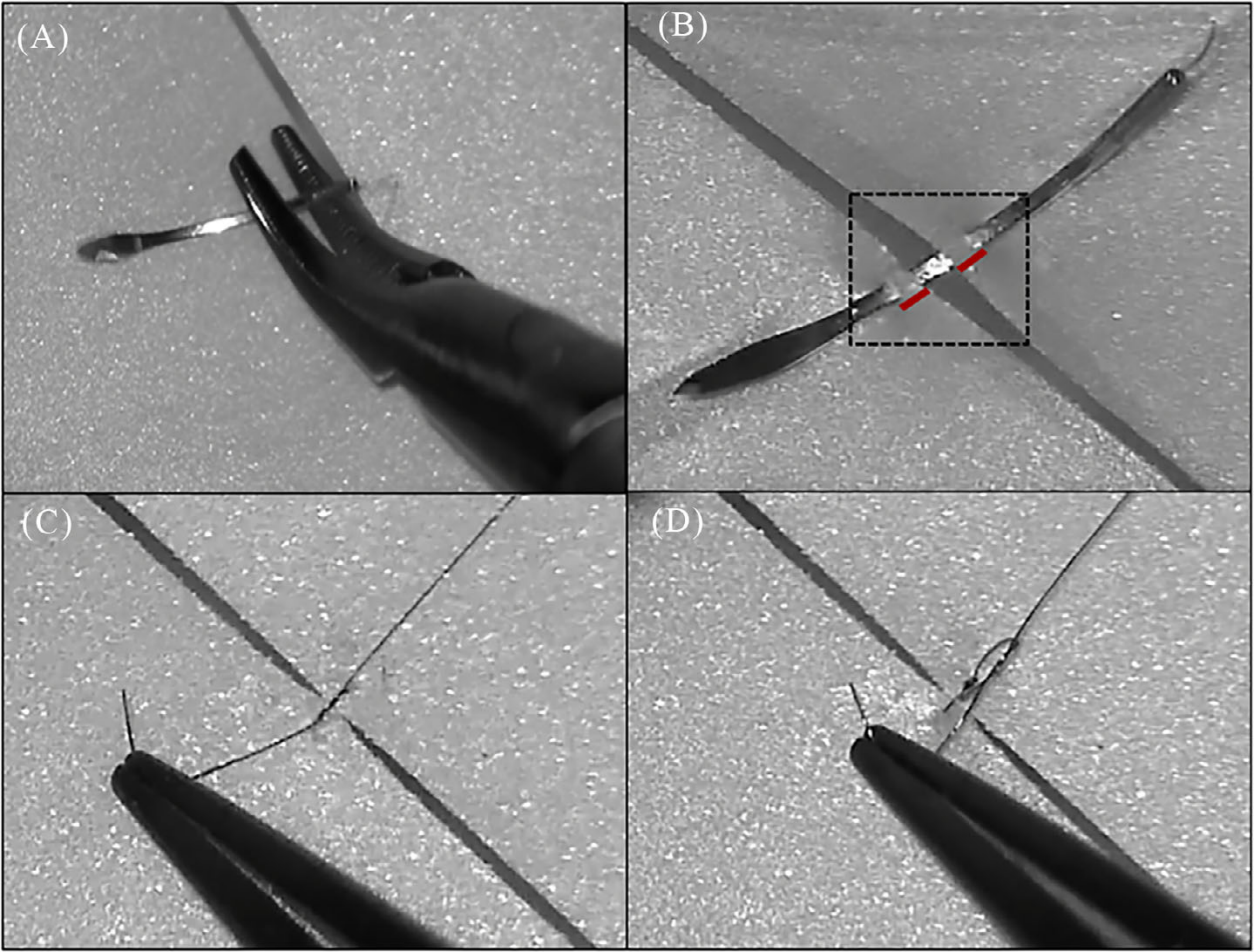
Breaking skills into smaller components. Suturing with microsurgical needles and threads under an operating microscope can be deconstructed into smaller, manageable components: (A) picking up the needle, (B) establishing symmetrical needle entry and exit points (see dashed outline), (C) edge approximation, and (D) knot construction.

### Repetition

Consistent and deliberate repetition of fundamental skills fosters automaticity, allowing these actions to become second nature. This process frees up valuable mental capacity (short‐term memory), enabling individuals to engage in higher‐order thinking and unlock their creative potential. With this automaticity in place, trainees can shift their focus to the strategic and analytical dimensions of their assigned tasks, enhancing both their efficiency and effectiveness.^19^ An excellent example that highlights this concept is the development of muscle memory during bimanual instrumentation‐guided suturing. Once this skill becomes second nature, it allows the microsurgeons to shift their focus to other critical aspects of wound closure, such as ensuring passive soft tissue approximation, achieving effective hemostasis, and maintaining optimal tissue hydration.

The effectiveness of deliberate practice hinges more on the quality of repetitions than on their sheer quantity. Engaging in high‐quality repetitions that target specific areas for growth leads to meaningful improvement, whereas aimless repetitions without a clear objective yield far less progress.

Directing attention to a specific task not only enhances the efficiency of time and effort but also ensures that every moment of practice contributes to measurable progress. Striving for intentional precision in every repetition amplifies skill refinement and accelerates development. Because these sessions require full engagement of both body and mind, they are often shorter in duration due to the natural fatigue that accompanies such intense focus and effort.

## CHARACTERISTICS OF DELIBERATE PRACTICE

### Purposeful and systematic

The development of skills requires a clear definition of objectives, strategically sound methods, and assessment of continuous improvement based on intentional actions.

### Focused on weaknesses

Contrary to naïve practice pursuits, deliberate focus on identifying and addressing shortcomings and performance inadequacies to eradicate them and mitigate their negative impact. Self‐awareness of vulnerabilities is essential in developing a systematic approach towards turning those limiting difficulties into strengths.

### Requires full attention

Complete and undivided focus and attention are required. Distractions must be avoided so every action becomes intentional in the pursuit of efficient skill refinement.

### Aims for constant improvement

Sustained dedication is essential to avoid plateauing and becoming stagnant in the process of skill improvement in microsurgery.

An example that encompasses the four characteristic elements of deliberate practice described above can be illustrated in the mechanics involved in picking up a microsurgical needle and efficiently securing it in the needle holder jaws so the suturing process can begin. Since the diameter of the microsurgical sutures is small (50–10μ),^20^ handling these delicate materials with microsurgical instruments specifically designed for such tasks enables precise visual‐manual proprioceptive feedback. This allows the practitioner to skillfully grasp the suture thread, gently lift and rotate the attached micro needle, and seamlessly transfer it to the needle holder. The needle's position can then be meticulously adjusted to orient it at the optimal angle, ensuring efficient and accurate tissue penetration (Video 3).

**VIDEO 3:** Picking up a micro needle. By securely grasping the suture thread, surgeons can delicately elevate and rotate the attached microneedle, facilitating its transfer to the needle holder. This maneuver enables precise positional adjustments of the needle, allowing optimization of its angular alignment. Such meticulous orientation ensures both efficient penetration and enhanced accuracy during tissue entry. See accompanying video file.

## APPLYING DELIBERATE PRACTICE

### Setting specific, measurable goals

Applying deliberate practice as a component for growth and continuous improvement of skills in the field of periodontics and implant dentistry requires transforming abstract aspirations into actionable, trackable goals. Once weaknesses are identified, implementing designed activities to remediate shortcomings would be fundamental to making progress in mastering skills. For example, if a microsurgeon demonstrates difficulty in achieving symmetric needle entry and exit points, a patterned rubber dam marked with indelible ink can be used to pre‐measure and label these points, ensuring a symmetrical distance between them. This visual guidance creates a mental model of equidistant landmarks, improving precision during needle manipulation (Video 4).

**VIDEO 4:** Entry/exit needle. Two equidistant points have been marked in black ink on a patterned rubber matrix. One extra point is marked in red ink. The goal is to enter through one black point and exit on the opposite black point. Then “park” the needle on the red point. Practicing parking the needle is useful to avoid having a loose needle in the oral cavity and to facilitate grabbing it again to proceed with the next suture. Rotating the rubber matrix so different working angles are experienced is a must. If this is too easy, try holding the needle holder with the non‐dominant hand. See accompanying video file.

### Breaking skills into smaller components

When mastering a complex procedure like suturing with microsurgical needles and threads under an operating microscope, it is advantageous to deconstruct the task into smaller, manageable components: 1) picking up the needle, 2) establishing symmetrical needle entry and exit points, 3) approximating the tissue, and 4) constructing knots. By concentrating on executing each step efficiently and accurately, one can achieve incremental successes that enhance confidence and solidify commitment to the overall process (Figure 3).

### Developing a practice plan

Carving out dedicated time for practice is paramount. Clinical execution of procedures cannot substitute for the intentional development of skills and mental models. Instead, deliberate practice must be purposefully scheduled, following a structured plan crafted by the microsurgeon and guided by an instructor or mentor.

### Seeking expert guidance or mentorship

Intrinsic motivation is crucial but insufficient for sustaining deliberate practice. To maximize effectiveness, expert guidance must be systematically integrated into the framework through adaptable methods such as individual coaching, group sessions, and/or mentorship. A mentor fosters holistic, long‐term development in a mentee‐driven, non‐evaluative relationship, offering personalized guidance and advice. In contrast, a coach focuses on short‐term, coach‐driven, and evaluative skill or performance improvement, using a standardized, question‐based approach.^21^ These structured support systems elevate skill development by transforming practice into a rewarding cycle of learning, refinement, and mastery. A coach offers insights into validated techniques and strategies for applying microsurgical principles to patient care. An optimized feedback loop is crucial for identifying strengths, addressing weaknesses, and refining task execution. Expert‐guided performance assessments help establish routines and patterns that serve as essential tools for high‐quality self‐evaluation.

## BENEFITS OF DELIBERATE PRACTICE

### Accelerated skill development

Deliberate practice offers a scientifically validated path to rapid skill mastery. By combining structured, goal‐oriented training with expert coaching, learners can accelerate expertise development in any field. Targeted challenges that push boundaries incrementally, continuous expert feedback for precise refinement, and iterative improvement cycles focusing on weaknesses are key elements of this approach, which outperforms casual repetition, making it particularly effective for those seeking optimal learning efficiency in new disciplines. The framework's emphasis on measurable progress and adaptive difficulty ensures sustained growth toward professional‐level competence.^22^, ^23^

### Overcoming plateaus

Growth stagnation and burnout often share a bidirectional relationship. Declining performance or failure to improve despite effort can lead to anxiety, goal abandonment, and negative impacts on patient care, team dynamics, and personal well‐being. Deliberate practice offers a solution by replacing repetitive routines with structured, goal‐oriented activities. Breaking skills into components, setting incremental goals, seeking mentorship for feedback, using varied simulators to avoid monotony, and incorporating rest between focused practice sessions can enhance skills and overcome professional plateaus. This approach optimizes individual potential and skill deployment.

## CHALLENGES AND LIMITATIONS

### Time‐consuming nature

Engaging in deliberate practice requires time allocation separate from the hours dedicated to patient care. Expert‐guided identification of weaknesses will assist in the preparation of simulators to support exercises designed to overcome those shortcomings. Strategic planning ahead will maximize efficiency and optimize the return on time spent setting up the space for training and executing the chosen drills.

### Mental and physical demands

Maintaining sustained focus while consistently pushing beyond comfort zones to optimize performance presents significant mental and physical challenges. Mitigating physical fatigue—which can impair both cognitive and motor functions—requires ergonomic accommodations such as neutral posture positioning and proper arm support during work with the microscope.^24^ Focused attention on measurable goals risks frustration, while visual modeling demands significant cognitive and emotional effort. Embracing discomfort is essential, as mastery often leads to progression into new challenges that demand full engagement. Intensive skill practice harnesses neuroplasticity, strengthening neural pathways for the precise motor control vital to microsurgical expertise.^25^, ^26^

### Potential for burnout

Rigorous activities associated with deliberate practice are demanding and require a healthy balance between rest and activity for proper recovery. If this balance is not observed, risk for burnout can occur. While skill decay is to be minimized by adhering to deliberate practice daily to weekly schedules, resting periods are not to exceed 1–2 weeks.^27^, ^28^ Incorporation of rest periods will help mental consolidation of skills.^29^, ^30^

## CONCLUSION

This manuscript summarizes the importance of the incorporation of deliberate practice as a mechanism to develop and enhance skills when working with the operating microscope.

It has identified its essential components (structured approach, its effortful and challenging nature, continues feedback, and repetition), defined its characteristics (purposeful and systematic, focused on weaknesses, demanding of full attention, and focus on constant improvement) and gives specific steps on applying this practice approach (setting specific‐measurable goals, breaking skills into smaller components, developing a practice plan, and seeking expert guidance). Lastly, it describes its benefits (accelerated skill development and overcoming plateaus) and limitations (time‐consuming, mental and physical demands, and potential for burnout).

With this layout, both the novice, advanced, and expert microsurgeon in periodontics and implant dentistry are given a tool to continue fine‐tuning skills and mastering this operational approach during the span of their careers.

## AUTHOR CONTRIBUTIONS

Diego Velásquez‐Plata wrote the original draft. Both authors discussed the structure of the paper, reviewed, edited, and approved the final manuscript.

## CONFLICT OF INTEREST STATEMENT

The authors declare no conflicts of interest.

## FOOTNOTE

1 Micro Trainer, Digital Surgicals Pte Ltd, Singapore.
